# Molecular characterization of immune responses of *Helicoverpa armigera* to infection with the mermithid nematode *Ovomermis sinensis*

**DOI:** 10.1186/s12864-019-5544-1

**Published:** 2019-02-27

**Authors:** Gui-Jie Wang, Xiao-Rong Zhuo, Wen-Wen Wang, Xu-Sheng Liu, Guo-Xiu Wang, Jia-Lin Wang

**Affiliations:** Hubei Key Laboratory of Genetic Regulation and Integrative Biology, School of Life Sciences, Central China Normal University, Wuhan, 430079 China

**Keywords:** *Ovomermis sinensis*, *Helicoverpa armigera*, Immunity, Parasitism, Fat body

## Abstract

**Background:**

Mermithid nematodes, such as *Ovomermis sinensis*, display a broad host range including some lepidopteran pests. Infective juveniles penetrate their host through the cuticle, complete their growth within the hemocoel and eventually kill the host upon their emergence. Hence, mermithid nematodes are considered potential biological control agents of insect pests. Our previous data indicate that the infection rate of *O. sinensis* on cotton bollworm (*Helicoverpa armigera*) is low, which may be largely due to the strong immune system of the host. However, current knowledge on the interactions of mermithid nematodes with their hosts and the mechanisms employed by hosts to defend themselves against mermithid nematodes is limited.

**Results:**

Here, we investigated the response of *H. armigera* to *O. sinensis* infection. Parasitism by *O. sinensis* caused a sharp decline in the survival rate of *H. armigera*. The hemocytic phagocytosis ability, antibacterial activity, and phenoloxidase (PO) activity in plasma of *H. armigera* increased at 1 d post parasitism (dpp) but decreased at 3 dpp. Further, we investigated gene expression in the fat body of parasitized and non-parasitized *H. armigera* larvae at 1, 3, and 5 dpp using a digital gene expression system. In total, 41, 60 and 68 immune-related differentially expressed genes were identified at 1, 3, and 5 dpp, respectively. These genes encoded pattern recognition receptors (PRRs), antimicrobial peptides (AMPs), serine proteases (SPs), SP inhibitors, mucins and other immune-related proteins. The expression of most *PRRs*, *AMPs*, *SPs*, and *mucins* was upregulated in the fat body of larvae at 1 dpp, downregulated at 3 dpp, and then again upregulated at 5 dpp by *O. sinensis*. The increased expression of SP inhibitors may contribute to the inhibited PO activity at 5 dpp.

**Conclusions:**

This study demonstrates that parasitism by *O. sinensis* modulates the immune reaction of the host *H. armigera* by altering the expression of immune-related genes. Our data provide a basis for future investigation of the molecular mechanisms employed by the mermithid nematode *O. sinensis* to modulate the immunity of the host *H. armigera*. These data will also likely facilitate the improvement of success in parasitism of *H. armigera* by *O. sinensis*.

**Electronic supplementary material:**

The online version of this article (10.1186/s12864-019-5544-1) contains supplementary material, which is available to authorized users.

## Background

Insects are one of the most successful species on earth. They occupy diverse habitats and survive under different biotic and abiotic stresses. To combat microbial or parasitic infections, insects have evolved cellular and humoral immune responses, which are initiated rapidly following the recognition of pathogens by pattern recognition receptors (PRRs). Cellular immune responses involve hemocyte-mediated encapsulation, phagocytosis, and nodulation, whereas humoral immune responses mainly involve the induction of antimicrobial peptides (AMPs) and prophenoloxidase (PPO) activating system [1, 2].

Entomopathogenic nematodes (EPNs) naturally infect and kill insects, thus representing a crucial alternative to chemicals for pest control [3, 4]. Nematodes employ different strategies to survive within the host insect, either by evading or suppressing the host immune response. EPNs belonging to the families Heterorhabditidae and Steinernematidae are well studied and widely used for pest control [3, 5]. Infective juveniles of the nematodes release symbiotic bacteria into the hemocoel after entering the insect host, thus killing the host within 24 to 48 h. Virulence factors such as toxin complexes, lipases, proteases, and lipopolysaccharides produced by symbiotic bacteria suppress host immune response [6, 7].

Despite impressive advances in the immune response of insects to microbial infection, our understanding of the molecular mechanisms involved in host–nematode interactions and host antiparasitic immune reactions remains obscure. Some studies suggest that nematodes modulate the host immune system via symbiotic bacteria. For example, Heterorhabditid nematodes are either not recognized or less efficiently recognized by the host immune system, and the induced PRRs of the host are response to the symbiotic bacteria of nematodes [8]. Similarly, the transcription of several *AMP* genes is activated in *Manduca* and *Drosophila* larvae upon infection with *Heterorhabditis* associated with the symbiotic bacteria *Photorhabdus* but not upon infection with axenic worms lacking the symbiotic bacteria [8, 9]. Other studies indicate that nematodes alone alter the transcription of certain immune-related genes. For example, infection of axenic *Heterorhabditis* nematodes results in the induction of several immune-related genes in adult flies [10]. Transcripts of several *PRRs*, *serine proteases* (*SPs*), *SP inhibitors* (*serpins*), and *AMPs* vary in *Armigeres subalbatus* mosquitoes infected with the filarial parasite *Brugia malayi* [11].

The developing stages of mermithid nematodes are parasitic, whereas the adults are free-living. Infective juveniles parasitize their host by penetrating through the cuticle. Once parasitism is established, the juveniles complete their growth inside the host, typically reaching impressive sizes. The juveniles eventually kill the host upon their emergence, suggesting the potential of mermithids for the biocontrol of insect pests. *Ovomermis sinensis*, a mermithid nematode, has been reported to display a broad host range, including lepidopteran pests such as the cotton bollworm (*Helicoverpa armigera*) [12]. Li et al. (2009) have demonstrated that parasitism with *O. sinensis* changes the spreading behavior of hemocytes and suppresses hemocytic encapsulation abilities of *H. armigera* [13]. We have previously shown that *H. armigera* C-type lectin 3 (CTL3) binds to the surface of *O. sinensis* and contributes to antiparasitic immune response [14].

However, the interactions of mermithid nematodes with their hosts and the immune response of the host to infection are poorly understood. In the present study, we evaluated the effect of parasitism by *O. sinensis* on the survival, phagocytosis ability, antibacterial activity, and PO activity of *H. armigera*. Further, gene expression dynamics in the fat body of parasitized and non-parasitized *H. armigera* larvae were investigated, and differentially expressed immune-related genes were obtained. These data improve our understanding of host–nematode interactions, and provide a comprehensive resource for exploring the molecular mechanism employed by the mermithid nematode *O. sinensis* to modulate the immune system of *H. armigera*.

## Results

### Effect of parasitism by *O. sinensis* on *H. armigera* survival

**Fig. 1:**
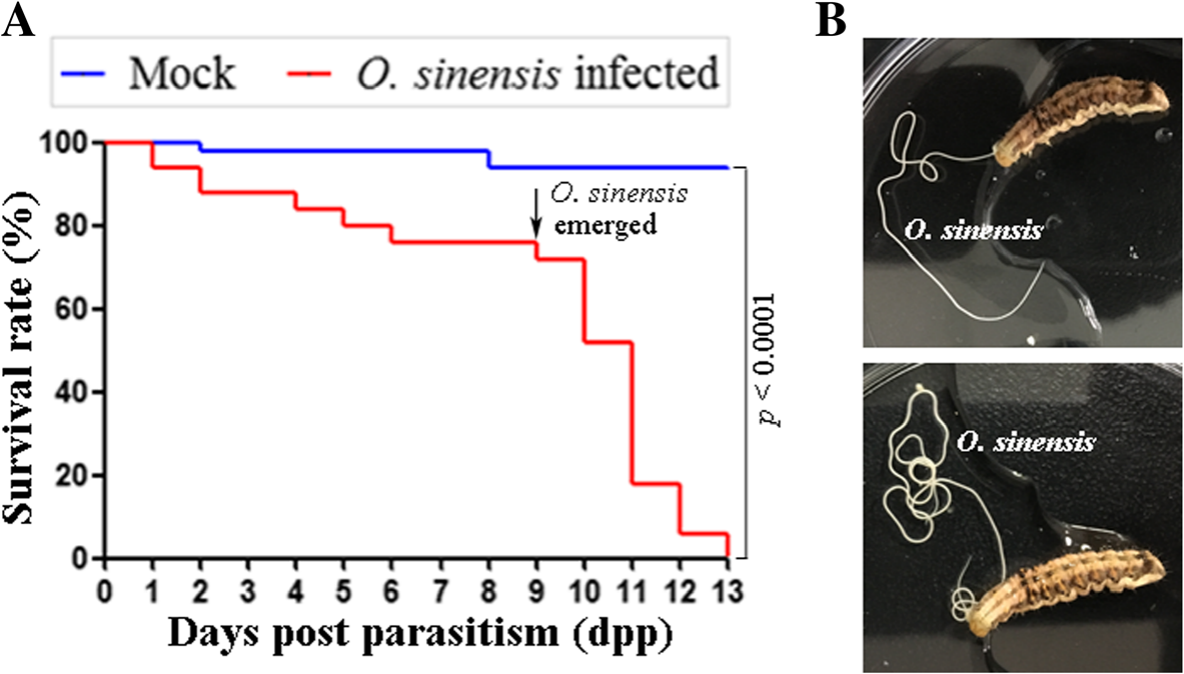
Parasitism by *Ovomermis sinensis* decreased *Helicoverpa armigera* survival. (**a**) Survival curves of *H. armigera* larvae infected with *O. sinensis* (red line; *n* = 50) in comparison with non-parasitized larvae (control; blue line; *n* = 50). *O. sinensis* emergence started at 9 d post parasitism (dpp), causing a sharp decline in the survival rate of *H. armigera* larvae. Significant difference (*p* < 0.0001, Log-rank test) was observed between *O. sinensis*-infected larvae and control groups. (**b**) *O. sinensis* emerged partially (upper panel) or completely (lower panel) from *H. armigera* larva

To evaluate the effect of *O. sinensis* on the pest control, the survival rate of *H. armigera* larvae parasitized by *O. sinensis* was investigated. The survival rate of parasitized larvae was much lower than that of the mock group, and all infected larvae died within 13 d post parasitism (dpp) (Fig. 1A). Juveniles of *O. sinensis* initially got fully developed at 9 dpp and emerged through the integument of the host, thereby killing the larvae (Fig. 1B). During the emergence of *O. sinensis* from 9 to 13 dpp, the parasitized group of *H. armigera* remained sixth-instar larvae while the mock group was pupae.

### Effect of parasitism by *O. sinensis* on *H. armigera* immune response

**Fig. 2:**
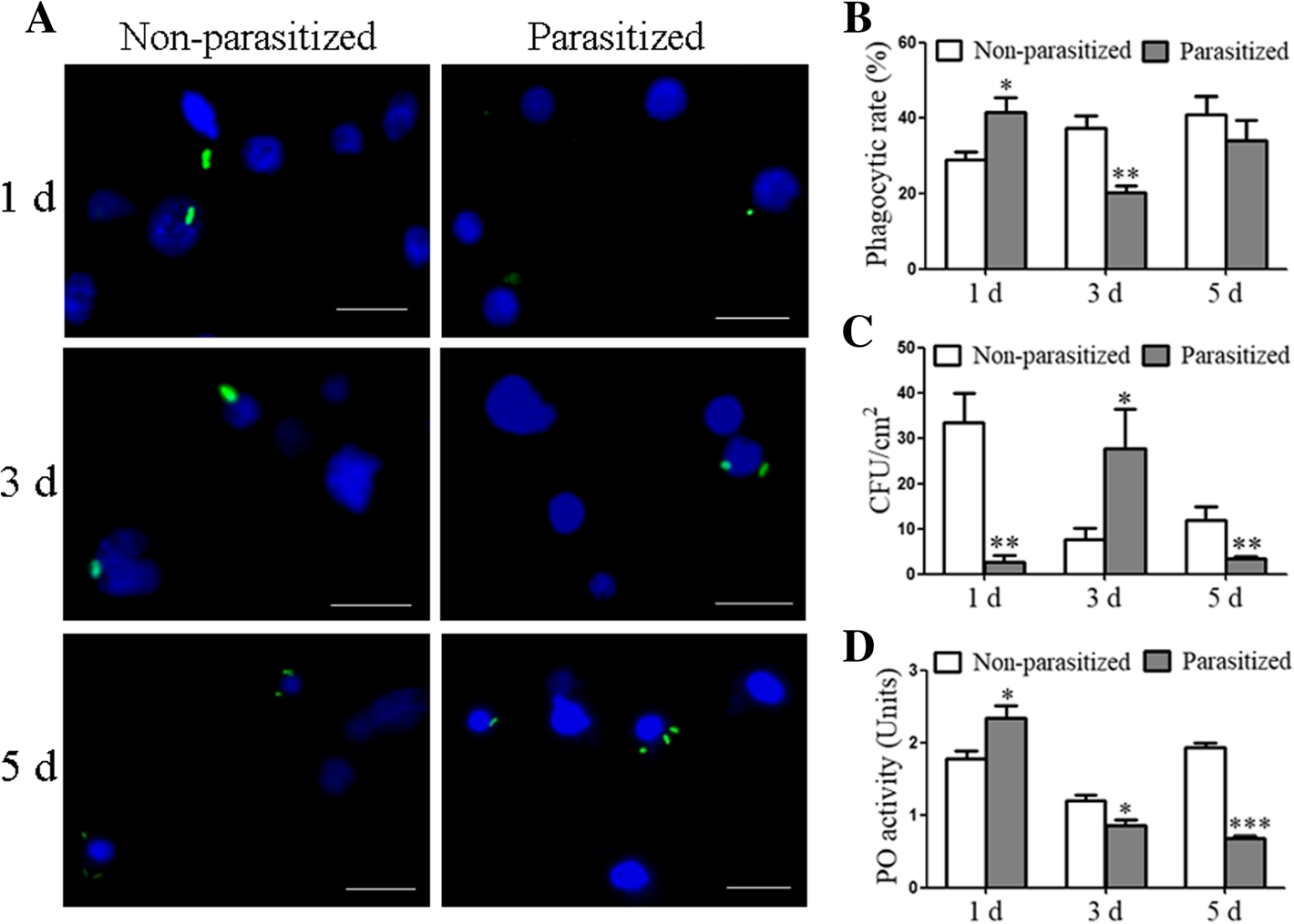
Dynamic immune responses of *H. armigera* larvae infected with *O. sinensis*. (**a**) Representative images of phagocytosis of *Escherichia coli*. (**b**) Statistical analysis of phagocytic rate. Hemocytes were collected from parasitized and non-parasitized *H. armigera* larvae at 1, 3, and 5 dpp. FITC-labeled *E. coli* (green) were subjected to phagocytosis analyses. DAPI was used to label hemocytic nucleus (blue). Scale bar = 10 μm. (**c**, **d**) Changes in antibacterial activity (**c**) and PO activity (**d**) in the plasma of *H. armigera* larvae. Plasma were collected from parasitized and non-parasitized larvae at 1, 3 and 5 dpp. Data represent mean ± standard error of mean (SEM) for three biological replicates. Significant differences were determined using Student′s *t*-test and are indicated with asterisks (* 0.01 < *p* < 0.05, ** 0.001 < *p* < 0.01, *** *p* < 0.001)

To test whether *O. sinensis* modulates the host immune response, we compared phagocytosis, antibacterial activity, and PO activity in plasma between parasitized and non-parasitized *H. armigera* larvae. The results showed that hemocytic phagocytosis of *Escherichia coli* was enhanced at 1 dpp and then decreased at 3 dpp, with no significant differences at 5 dpp (Fig. 2A and B). Antibacterial activities in the plasma were promoted at 1 and 5 dpp and reduced at 3 dpp (Fig. 2C). Plasma PO activity was slightly increased at 1 dpp, and then significantly reduced at 3 and 5 dpp (Fig. 2D). Since parasitism by *O. sinensis* enhanced phagocytosis and antibacterial activity in host at 1 dpp, we wonder whether *O. sinensis* was associated with bacteria. PCR analyses indicated that *O. sinensis* juveniles harbored bacteria (Additional file 1: Figure S1).

### RNA-Seq analysis and identification of differentially expressed transcripts (DETs)

To explore the molecular mechanism employed by *O. sinensis* to alter host immunity, we compared digital gene expression (DGE) profiles in the fat body of parasitized (FP) and non-parasitized (control; FC) *H. armigera* larvae. FP and FC were dissected from larvae at 1 (FP1, FC1), 3 (FP3, FC3), and 5 (FP5, FC5) dpp, and three biological replicates were performed at each time point. Parasitism by *O. sinensis* delays *H. armigera* larval development. FP1 and FC1 represent fat bodies both of fifth-instar larvae at feeding stage. FP3 and FC3 correspond to fat bodies of fifth-instar larvae at head capsule slippage (HCS) and sixth-instar larvae at feeding stage, respectively. FP5 and FC5 represent fat bodies of sixth-instar larvae at feeding stage and metamorphic stage, respectively. In total, 18 RNA-Seq libraries (FP1–1, − 2, − 3; FC1–1, − 2, − 3; FP3–1, − 2, − 3; FC3–1, − 2, − 3; FP5–1, − 2, − 3; FC5–1, − 2, − 3) were constructed and sequenced. The number of raw reads generated from each library ranged from 23.96 to 27.92 million, whereas the number of cleaned reads ranged from 21.01 to 23.62 million. Cleaned reads from each library were mapped to previously assembled unigenes of *H. armigera* fat body [15], with a mapping ratio ranging from 49.36 to 58.76% (average 52.91%) (Additional file 2: Table S1).

To compare the differential gene expression profiles between FP and FC larvae at different time points (FP1 vs. FC1; FP3 vs. FC3; FP5 vs. FC5), fragments per kilobase of transcript per million mapped fragments (FPKM) were quantified. Heatmap indicated that the square of correlation coefficient ranged from 0.909–0.99, 0.975–0.993, 0.994–0.996, 0.871–0.977, 0.978–0.99, and 0.912–0.99 in FC1, FC3, FC5, FP1, FP3, and FP5 groups, respectively (Additional file 3: Figure S2). These high Pearson values suggest the reliable replications and the reasonable samples.

**Fig. 3:**
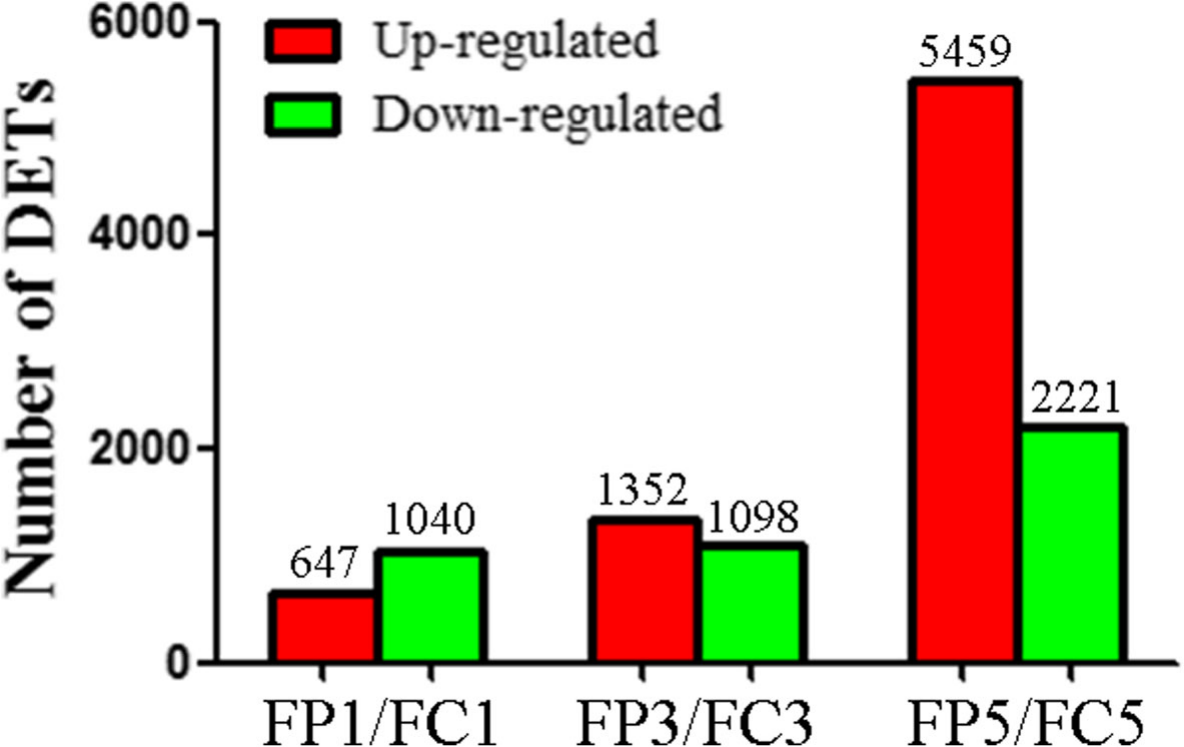
Comparisons of differentially expressed transcripts (DETs) between the FP1 and FC1, FP3 and FC3, and FP5 and FC5. The FP1, FP3, and FP5 represent the fat body of *H. armigera* larvae parasitized by *O. sinensis* at 1, 3, and 5 dpp, respectively. The FC1, FC3, and FC5 represent the fat body of non-parasitized larvae (controls) collected at the same time points. The number of upregulated DETs is indicated in the red column, whereas the number of downregulated DETs is represented in green

NOISeq-sim was adopted to screen DETs with cut-off thresholds of |log2 (FP/FC ratio)| ≥ 1 and divergent probability ≥0.8. A total of 1687 DETs were identified from the FP1 vs. FC1, of which 647 were upregulated and 1040 were downregulated. A total of 1352 DETs were upregulated and 1098 DETs were downregulated from the FP3 vs. FC3, whereas 5459 DETs were upregulated and 2221 DETs were downregulated from the FP5 vs. FC5 (Fig. 3 and Additional file 4: Table S2).

### Expression dynamics of immune-related genes in the parasitized fat body

**Fig. 4:**
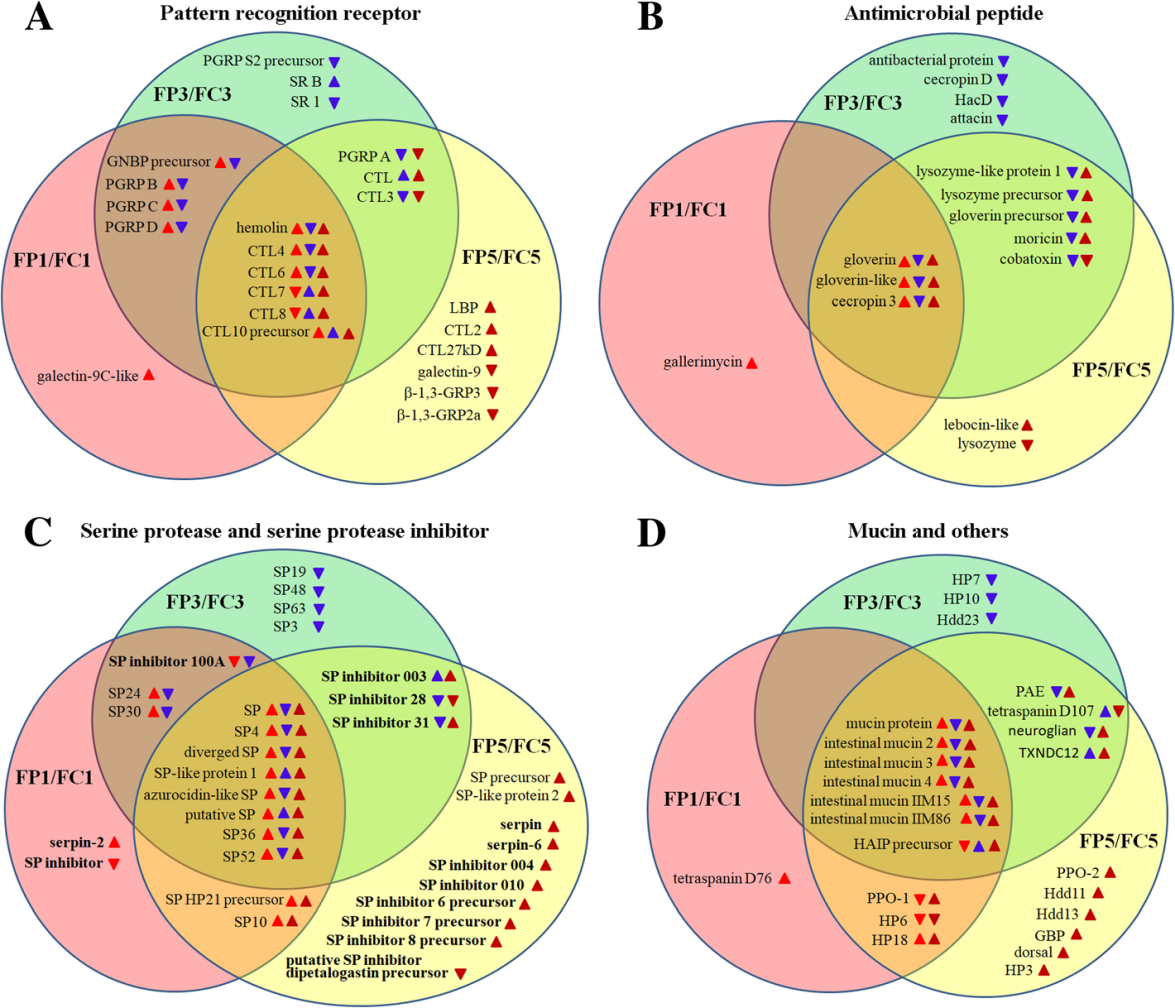
Schematic representation of common and unique immune-related differentially expressed genes identified based on comparisons between FP and FC samples. (**a**) *Pattern recognition receptors* (*PRRs*). (**b**) *Antimicrobial peptides* (*AMPs*). (**c**) *Serine proteases* (*SPs*) and *SP inhibitors* (*serpins*). (**d**) *Mucins* and others. The FP1, FP3, and FP5 represent the fat body of *H. armigera* larvae parasitized by *O. sinensis* at 1, 3, and 5 dpp, respectively, whereas FC1, FC3, and FC5 represent the fat body of non-parasitized larvae (controls) at the same time points. Genes in overlapping regions were identified as differentially expressed in two or three groups. Red, blue, and purple arrows represent expression level changes in FP1 vs. FC1, FP3 vs. FC3, and FP5 vs. FC5, respectively. Upward- and downward-pointing arrows represent the directions of expression level changes

Of the 1687 DETs identified from the FP1 vs. FC1 comparison, 80 DETs representing 41 genes were involved in immunity; these encoded 11 PRRs, 4 AMPs, 12 SPs, 3 SP inhibitors, 6 mucins and 5 others. Of the 2450 DETs identified from the FP3 vs. FC3 comparison, 131 DETs representing 60 genes were involved in immunity, including 16 PRRs, 12 AMPs, 14 SPs, 4 SP inhibitors, 6 mucins and 8 others. Of the 7680 DETs identified from the FP5 vs. FC5 comparison, 209 DETs representing 68 genes were involved in immunity, including 15 PRRs, 10 AMPs, 12 SPs, 11 SP inhibitors, 6 mucins, and 14 others (Fig. 4 and Additional file 5: Table S3).

**Fig. 5:**
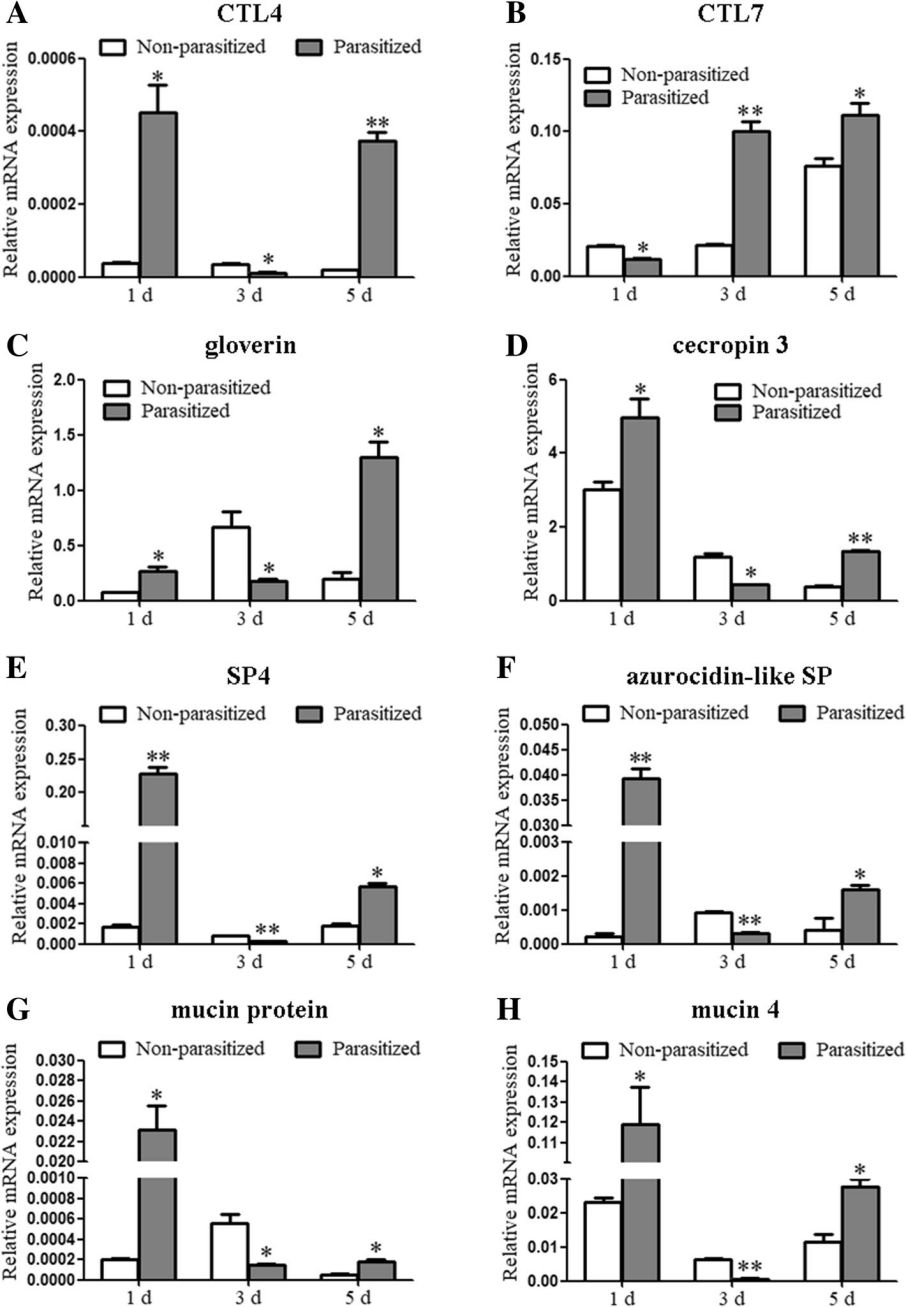
Validation of RNA-Seq data using qRT-PCR analysis. **a** *CTL4*. **b** *CTL7*. **c** *gloverin*. **d** *cecropin 3*. **e** *SP4*. **f** *azurocidin-like SP*. **g** *mucin protein*. **h** *mucin 4*. Fat bodies were dissected from parasitized and non-parasitized *H. armigera* larvae, and the expression level of several immune-related genes was analyzed using qRT-PCR. All expression values are calculated against the reference gene *β-actin*. Data represent mean ± SEM of three biological replicates. Significant differences were determined using Student’s *t*-test and are indicated using asterisks (* 0.01 < *p* < 0.05, ** 0.001 < *p* < 0.01)

Most of the *PRR*, *AMP*, *SP,* and *mucin* genes were upregulated in parasitized larvae at 1 dpp, downregulated at 3 dpp and again upregulated at 5 dpp, compared with non-parasitized larvae. Two *PRRs* (*CTL4*, *CTL7*), two *AMPs* (*gloverin*, *cecropin 3*), two *SPs* (*SP4*, *azurocidin-like SP*), and two *mucins* (*mucin protein*, *mucin 4*), which were shared among FP1 vs. FC1, FP3 vs. FC3, and FP5 vs. FC5 groups, were selected for quantitative real-time PCR (qRT-PCR) analyses. The results of qRT-PCR validated RNA-Seq results (Fig. 5). The expression of most *SP inhibitors* (*serpins*) was upregulated in parasitized larvae at 5 dpp, compared with non-parasitized larvae.

### Expression of *PRR* genes

Four differentially expressed *PRRs* [*Gram negative binding protein* (*GNBP*) *precursor*, *peptidoglycan recognition protein B* (*PGRP B*), *PGRP C*, and *PGRP D*] were shared between FP1 vs. FC1 and FP3 vs. FC3 comparisons, all of which were upregulated in FP1 vs. FC1 and downregulated in FP3 vs. FC3. The *PGRP A* and *CTL3* genes were downregulated, whereas *CTL* was upregulated in both FP3 vs. FC3 and FP5 vs. FC5 comparisons. No overlap in the *PRRs* that showed differentially expressed between FP1 vs. FC1 and FP5 vs. FC5. Additionally, six differentially expressed *PRRs* (*hemolin*, *CTL4*, *CTL6*, *CTL7*, *CTL8*, and *CTL10 precursor*) were shared among FP1 vs. FC1, FP3 vs. FC3, and FP5 vs. FC5; the expression of *hemolin*, *CTL4* and *CTL6* was first increased in FP1 vs. FC1, decreased in FP3 vs. FC3, and then again increased in FP5 vs. FC5 (Fig. 4A).

### Expression of *AMP* genes

Five differentially expressed *AMPs* (*lysozyme-like protein 1*, *lysozyme precursor*, *gloverin precursor*, *moricin*, and *cobatoxin*) were shared between FP3 vs. FC3 and FP5 vs. FC5. No overlap in the *AMP* transcripts that showed differentially expressed either between FP1 vs. FC1 and FP3 vs. FC3 or between FP1 vs. FC1 and FP5 vs. FC5. Of the differentially expressed *AMP* genes, three (*gloverin*, *gloverin-like* and *cecropin 3*) were shared among FP1 vs. FC1, FP3 vs. FC3, and FP5 vs. FC5. All of *AMP* genes identified were upregulated in FP1 vs. FC1, downregulated in FP3 vs. FC3 and upregulated in FP5 vs. FC5, with the exception of *cobatoxin* and *lysozyme*, which were downregulated in FP5 vs. FC5 (Fig. 4B).

### Expression of *SP* and *SP inhibitor* genes

Two differentially expressed *SP* genes (*SP24*, *SP30*) and one differentially expressed *SP inhibitor* gene (*SP inhibitor 100A*) were shared between FP1 vs. FC1 and FP3 vs. FC3. Two additional differentially expressed *SPs* (*SP HP21 precursor*, *SP10*) were shared between FP1 vs. FC1 and FP5 vs. FC5, and three differentially expressed *SP inhibitors* (*SP inhibitor 003*, *28*, and *31*) were shared between FP3 vs. FC3 and FP5 vs. FC5. Among the differentially expressed *SP* genes, eight (*SP*, *SP4*, *diverged SP*, *SP-like protein 1*, *azurocidin-like SP*, *putative SP*, *SP36*, and *SP52*) were shared among FP1 vs. FC1, FP3 vs. FC3, and FP5 vs. FC5; the expression of all of these eight genes was upregulated in FP1 vs. FC1 and FP5 vs. FC5, whereas downregulated in FP3 vs. FC3 with the exception of *SP-like protein 1* and *putative SP*, which were upregulated. Additionally, nine *SP inhibitor* genes (*serpin*, *serpin-6*, *SP inhibitor 003*, *004*, *010*, *6 precursor*, *7 precursor*, *8 precursor* and *31*) were upregulated in FP5 vs. FC5 (Fig. 4C).

### Expression of *mucin* and other genes

Six *mucin* genes [*mucin protein*, *intestinal mucin* (*IIM*) *2*, *3*, *4*, *IIM15*, and *IIM86*] were upregulated in FP1 vs. FC1, downregulated in FP3 vs. FC3, and again upregulated in FP5 vs. FC5. The abundance of *PPO-1* transcripts was inhibited in FP1 vs. FC1, whereas transcripts of *prophenoloxidase activating enzyme* (*PAE*) were suppressed in FP3 vs. FC3. The *PPO-1*, *PPO-2*, and *PAE* transcripts were increased in FP5 vs. FC5 (Fig. 4D).

## Discussion

Unlike EPNs (Heterorhabditidae and Steinernematidae) that kill their host within 24–48 h post infection, the mermithid nematode *O. sinensis* killed most of the *H. armigera* larvae within 9–13 dpp in this study. The efficient insecticidal effect of Heterorhabditidae and Steinernematidae is due to their symbiotic bacteria, which are released within the insect hemocoel and secrete virulence factors [6, 7]. Hence, this nematode-bacteria symbiosis is crucial for efficient action of EPNs in pest control [16]. The mermithid nematode *Strelkovimermis spiculatus* serves as a vector of the iridescent virus [17]; however, association of mermithid nematode with symbiotic bacteria has been rarely detected [18]. Although *O. sinensis* was associated with bacteria, we cannot conclude whether the bacteria was symbiotic or came from outside contamination. Considering that *O. sinensis* kills the host mostly by emerging through the host integument, which takes a relatively long time, it is likely that *O. sinensis* does not harbor pathogenic bacteria.

*O. sinensis* not only takes a long time to kill its hosts, but also exhibits a relatively low infection rate in the host *H. armigera*, as shown in our laboratory [14]. The low infection rate of *O. sinensis* on *H. armigera* may be largely due to the highly developed immune system of the host. Hence, clarification of the interactions between *O. sinensis* and its host, especially the immune response of *H. armigera* to *O. sinensis* is of vital importance. The relatively low mapping ratio of the cleaned reads to previously assembled unigenes of peptidoglycan (PGN)-challenged fat body was observed. This may reflect the differences in the transcriptional profiles between the *O. sinensis*-challenged fat body and the PGN-challenged one, or the insufficient sequencing depth of the previous fat body transcriptome [15]. The number of DETs between FP and FC increased along with larval development. These DETs represent a broad range of pathways, including metabolic pathways, fat digestion and absorption, fatty acid biosynthesis, steroid biosynthesis and Ribosome (data not shown). Here, we focused on molecular characterization of immune responses.

Insects employ hemocytic phagocytosis against bacteria, which requires the participation and coordination of both cellular and humoral factors. Several humoral PRRs such as opsonic factors, secreted mainly from fat body, function in non-self recognition and triggering phagocytosis [19, 20]. Since the phagocytosis ability of *H. armigera* is largely due to PRRs, which attach to hemocytes, variation in the expression levels of genes encoding PRRs at different time points post parasitism by *O. sinensis* would have important consequences. The expression of many *PRR* genes is consistent with the phagocytosis ability of *H. armigera*, as some PRR proteins are involved in phagocytosis. For example, PGRP D has previously been demonstrated to promote phagocytosis of *E. coli* in *H. armigera* [21]. In this study, the upregulation and downregulation of *PGRP D* expression at 1 and 3 dpp by *O. sinensis*, respectively, were consistent with the phagocytosis ability of *H. armigera* at these time points. Similar dynamic expression of *PRRs* has also been detected in *A. subalbatus* in response to *B. malayi* infection, with a *PGRP* transcript upregulated at 1 h post infection and a *CTL* transcript downregulated at 12 or 24 h post infection [11].

The EPN *Steinernema feltiae* modulates hemocytic phagocytosis by removing opsonic factors from the host hemolymph, thus supporting its symbiotic bacteria [22]. Since *O. sinensis* most likely does not need to support symbiotic bacteria, initial infection of *O. sinensis* is presumed to activate the host immune system, for example by activating phagocytosis. In addition to the role of PRRs in phagocytosis, some PRRs also participate in encapsulation. For example, infection of *A. subalbatus* with the filarial worm *Dirofilaria immitis* leads to induction of β1,3-glucan recognition protein, a PRR involved in encapsulation [23]. Although the expression of most *PRR* genes was upregulated in *H. armigera* at 1 dpp by *O. sinensis*, encapsulation was suppressed possibly because of the destruction of hemocyte cytoskeleton, as demonstrated previously [13]. Parasitization also suppresses host cellular encapsulation by inhibiting the expression of PRRs such as CTL and scavenger receptor [24, 25]. Given that PGRP D and CTL3 have been previously demonstrated to promote encapsulation [14, 21], we speculate that the decreased expression of certain *PRRs* at 3 dpp would facilitate the survival of *O. sinensis* within the homocoel of *H. armigera*.

Antimicrobial effectors such as AMPs are mainly synthesized in fat body and subsequently secreted into the hemolymph, and play important roles in the restriction or elimination of the invading pathogen [26]. Infection of *Aedes aegypti* with *Wuchereria bancrofti* filarial nematodes has been shown to increase mRNA levels of *defensin*, *cecropin* and *transferrin* [27]. Some researchers argue that the upregulation of *AMP* transcripts may be attributed to symbiotic bacteria, considering it is symbiotic nematodes but not axenic nematodes that induced the transcription of *AMP* genes [8, 9]. Other studies have raised the possibility that the increased expression of immune-related genes (including *AMPs*) reflects strategies for tolerating tissue damage caused by nematodes [28]. Cecropin was reported to attenuate the motility of the filarial nematode *Brugia pahangi* in vitro and reduce the number of *B. pahangi* in vivo [29]. Hence, we speculate that the expression of *AMP* genes is increased in response to either *O. sinensis* or bacteria associated with *O. sinensis*, which needs further investigation.

To establish infection, nematodes must be capable of suppressing the immunity of their host. Nematodes modulate the humoral and cellular immune responses of the host by producing molecules such as proteases. For example, symbiotic *Heterorhabditis bacteriophora* secrete a proteinase into the bodies of greater wax moth larvae and inhibit the expression of *cecropin* [30]. In this study, we showed upregulation of *AMP* genes in *H. armigera* at 1 dpp by *O. sinensis* and downregulation at 3 dpp; this was consistent with the antibacterial activity in plasma. The *AMP* genes exhibit similar expression profiles in *A. subalbatus* at different time points post infection by filarial worm. For example, the expression of two *cecropin* genes is increased at 6 h post infection and that of two *defensin* and two *lysozyme* genes is decreased at 12 h post infection [11]. Since molecules produced by *O. sinensis* have not yet been characterized, the mechanisms employed by *O. sinensis* to suppress the immunity of the host remain unclear. Interestingly, we observed upregulation of *AMP* genes and enhanced antibacterial activity in *H. armigera* larvae at 5 dpp by *O. sinensis*, possibly because *O. sinensis* may have developed sufficiently during 5 d to endure higher immune stress. Mucin is reported to be involved in the entrapment of bacteria [31]. In this study, *mucin* genes exhibited a similar expression profile as the *AMP* genes; thus, it is likely that mucins function in antiparasitic immunity and contribute toward antibacterial activity in plasma.

SPs and SP inhibitors (serpins) play important roles in melanin biosynthesis, and are most likely involved in antiparasitic immunity [32, 33]. Global transcriptional response of *A. aegypti* to *B. malayi* infection has revealed an increase in the abundance of several *SP* transcripts [28]. Transcriptional levels of 30 *SP* or *serpin* genes vary in *A. subalbatus* at different time points following infection by *B. malayi* [11], suggesting a potential role of melanization in antiparasitic immunity. In this study, the abundance of most *SP* transcripts in *H. armigera* larvae parasitized by *O. sinensis* increased at 1 dpp and decreased at 3 dpp. Given that SP functions in the activation of PPO and is required for parasite melanization [34, 35], it is reasonable that PO activity in plasma slightly increased at 1 dpp and decreased at 3 dpp. However, at 5 dpp, the abundance of most of the *SP* transcripts increased, whereas PO activity decreased. This may be because of the increased abundance of *SP inhibitor* transcripts, as serpin inhibits the activation of PPO by SP [36].

Some virulence factors produced by parasites during infection protect them against the potent effects of the immune system of the host and improve the success rate of parasitism. For example, a trypsin-like serine protease and a chymotrypsin serine protease secreted during the parasitic phase of *Steinernema carpocapsae* exhibit PPO inhibitory activity [37, 38]. Here we showed that *O. sinensis* parasitism modulated PO activity of *H. armigera* larvae by altering the expression of *SPs* and *serpins*, although the underlying mechanisms remain unclear.

## Conclusions

Overall, our data revealed dynamic immune responses of *H. armigera* to *O. sinensis* infection. The initial infection of *H. armigera* by *O. sinensis* activated the expression of many *PRR*, *AMP*, *SP,* and *mucin* genes, which is consistent with the enhanced immune reactions of the host (phagocytosis, antibacterial activity, and PO activity). Subsequently, *O. sinensis* suppressed these immune reactions by inhibiting the expression of most *PRR*, *AMP*, *SP,* and *mucin* genes to facilitate its survival within the host. After the successful establishment of *O. sinensis* parasitism, we speculate that *O. sinensis* was able to endure the relatively higher immune-related stress (such as antibacterial activity) presumably because it has grown to a sufficiently large size within 5 dpp. However, the inhibited PO activity at 5 dpp may be attributed to the increased expression of *serpins*, suggesting that *O. sinensis* is more sensitive to melanization. We provide a comprehensive resource for exploring the complex molecular mechanisms underlying the interaction between the mermithid nematode *O. sinensis* and its host *H. armigera*. Further investigation of DEGs between FP and FC would provide critical target genes for improvement of infection rate. Characterization of virulence factors produced by *O. sinensis* and elucidation of the mechanism employed by *O. sinensis* to suppress host immunity will also be our future study. Our findings will likely facilitate the development of *O. sinensis* as an effective and eco-friendly biological control agent.

## Methods

### Culture conditions, PCR amplification and infection of *H. armigera* larvae

*H. armigera* larvae were maintained in the laboratory at 28 ± 1 °C, 70% relative humidity and 14 h light/10 h dark photoperiod. Larvae were reared on an artificial diet mainly made from wheat germ and soybean powder [39].

A colony of adult *O. sinensis* nematodes was collected from a wheat field in Shangcai, Henan, China. These nematodes were maintained in the laboratory until infective juveniles were obtained. Approximately one thousand juveniles were applied for genomic DNA extraction using a bacterial DNA kit (OMEGA, USA). The primers (Additional file 6: Table S4) were designed to amplify the V4-V5 region of 16S rRNA. Then PCR reactions were performed with 30 ng of genomic DNA as a template.

Fifteen juvenile nematodes were used to infect a fourth-instar larva of *H. armigera* for 3 h, as described previously [14]. After successful parasitization by *O. sinensis*, *H. armigera* larvae continued to feed on artificial diet until the emergence of fully developed nematodes. The number of dead larvae was recorded every day. Infected larvae, which died before the emergence of fully developed nematodes, were dissected to confirm whether the juveniles succeeded in penetrating. Survival curves were created and analyzed using GraphPad software. Log-rank (Mantel-Cox) test was performed to calculate statistical significance.

### Phagocytosis assay

Fluorescein isothiocyanate (FITC; Sigma) labeling of *E. coli* was conducted at 37 °C for 1 h. Samples were washed five times with PBS, and FITC-labeled bacteria were resuspended in PBS to a final concentration of 2 × 10^8^ cells/ml. Phagocytosis analyses were performed in triplicate, as described previously [40], with minor modifications. Briefly, hemocytes were collected from parasitized and non-parasitized larvae at various time points and suspended in PBS. Subsequently, PBS containing FITC-labeled bacteria was added to the PBS containing hemocytes. After incubation for 1 h, aliquots of the mixture were dispensed onto glass slides, and hemocytes were allowed to settle down for 30 min. Hemocytes were then fixed with 4% formaldehyde for 10 min, washed and then observed under a fluorescence microscope. The phagocytic rate was calculated as follows:

$$ \mathrm{Phagocytic}\ \mathrm{rate}\ \left(\%\right)=\left[\mathrm{Number}\ \mathrm{of}\ \mathrm{bacteria}\hbox{-} \mathrm{ingesting}\ \mathrm{hemocytes}/\mathrm{Total}\ \mathrm{number}\ \mathrm{of}\ \mathrm{hemocytes}\right]\times 100 $$

### Measurement of antibacterial activity

Hemolymph was collected from parasitized and non-parasitized larvae at various time points, and diluted 3-fold in sterile anticoagulant buffer. After centrifugation at 1000 rpm for 10 min, cell-free plasma was obtained, and antibacterial activity was determined as described previously [15]. Briefly, 90 μl of plasma was mixed with 10 μl of *E. coli* suspension. After incubation for 1 h at room temperature, the plasma–bacteria mixture was plated onto lysogeny broth agar plates and incubated at 37 °C overnight. Subsequently, the number of colony forming units (CFU) was counted in each plate.

### Measurement of PO activity

To evaluate the effect of parasitism by *O. sinensis* on PO activity of *H. armigera*, plasma was collected from parasitized and non-parasitized larvae at various time points. PO activity was measured as described previously [41]. Briefly, plasma (50 μl) was incubated with 50 μl trypsin (2 mg/ml) for 20 min at room temperature, followed by addition of 50 μl substrate solution containing dopamine (3 mg/ml). Initial absorbance was measured at 490 nm, and one unit of PO activity was defined as the amount of enzyme yielding an increase of 0.001 absorbance units per min.

### RNA extraction, DGE library preparation and RNA-Seq analysis

To prepare DGE libraries, three biological replicates of FP and FC samples were collected at 1, 3 and 5 dpp. Total RNA was extracted from these samples using TRIzol Reagent (Invitrogen), and used with oligo dT magnetic beads to enrich mRNAs. The mRNA samples were then fragmented into short sequences and reverse transcribed using N6 random primer. Subsequently, the resulting double-stranded complimentary DNA was end-repaired to generate blunt ends and ligated with two blunt end adaptors. Following PCR amplification, the PCR products were denatured, and single-stranded DNA was cyclized using splint oligo. The prepared libraries were subjected to SE50 sequencing at the Beijing Genomics Institute.

### Mapping reads to the reference unigenes and analysis of DETs

The high number of unknown bases, adaptor sequences and low quality reads were filtered from raw sequence reads to generate clean reads. Clean reads were then mapped onto reference sequences of *H. armigera* fat body transcriptome generated previously [15] using Bowtie2 [42]. To eliminate the influence of gene length and sequencing discrepancy, gene transcripts were quantified as FPKM values for comparing expression levels of DETs among samples. Correction for false positive and false negative errors were performed using false discovery rates (FDR), with FDR ≤ 0.001 as the default threshold to judge the significance of gene expression differences. Definition of divergence probability of each transcript differentially expressing was following the formula described previously [43]. Values of |log2 fold-change| ≥ 1 and divergence probability ≥0.8 were used as cut-off thresholds for identifying DETs between the different experimental conditions, based on a non-parametric algorithm NOISeq-sim [44].

### Validation of gene expression using qRT-PCR

Results of RNA-Seq analysis were validated using qRT-PCR. Total RNA (2 μg) prepared for RNA-Seq was used for the synthesis of first-strand cDNA. Subsequently, qRT-PCR was conducted with *TransStart* Top Green qPCR SuperMix (TransGen Bio-tech, Beijing, China) using a CF × 96 system (Bio-Rad, Hercules, CA, USA). A total of eight differentially expressed genes, including *PRRs* (*CTL4*, *CTL7*), *AMPs* (*gloverin*, *cecropin 3*), *SPs* (*SP4*, *azurocidin-like SP*), and *mucins* (*mucin protein*, *mucin 4*), were selected for qRT-PCR analyses. The expression level of each gene was normalized relative to that of the reference gene *β-actin* using the 2^-ΔCT^ method (ΔC_T_ = C_T[test gene]_ - C_T[β-actin]_). Gene-specific primers used for qRT-PCR analysis are listed in Additional file 6: Table S4.

## Additional files

Additional file 1: **Figure S1.** PCR analyses confirming the association of *O. sinensis* with bacteria. M, DL2000 DNA marker. Lane 1, PCR amplicon of V4-V5 region of 16S rRNA. (TIF 38 kb)

Additional file 2: **Table S1.** Statistics of DGE library sequencing and reads mapping. (XLS 20 kb)

Additional file 3: **Figure S2.** Heatmap indicating the square of correlation value from three biological replicates. The correlation values were assessed by using the Pearson method. (TIF 476 kb)

Additional file 4: **Table S2.** Transcripts differentially expressed between the fat body of parasitized and non-parasitized larva. (XLS 3723 kb)

Additional file 5: **Table S3.** Immune-related DETs between the fat body of parasitized and non-parasitized larva. (XLS 191 kb)

Additional file 6: **Table S4.** List of primers used. (XLS 21 kb)

## Abbreviations

AMPs: antimicrobial peptides
CFU: colony forming units
CTL: C-type lectin
DETs: differentially expressed transcripts
DGE: digital gene expression
dpp: d post parasitism
EPNs: entomopathogenic nematodes
FC: fat body of non-parasitized
FDR: false discovery rates
FITC: Fluorescein isothiocyanate
FP: fat body of parasitized
FPKM: fragments per kilobase of transcript per million mapped fragments
GNBP: Gram negative binding protein
HCS: head capsule slippage
PAE: prophenoloxidase activating enzyme
PGRP: peptidoglycan recognition protein
PO: phenoloxidase
PPO: prophenoloxidase
PRRs: pattern recognition receptors
qRT-PCR: quantitative real-time PCR
SPs: serine proteases
