# Supplementary figures and images for: Molecular characterization of immune responses of Helicoverpa armigera to infection with the mermithid nematode Ovomermis sinensis
Source: BMC Genomics. 2019 Feb 27;20:161. doi: 10.1186/s12864-019-5544-1 (PMC6391810; doi:10.1186/s12864-019-5544-1)

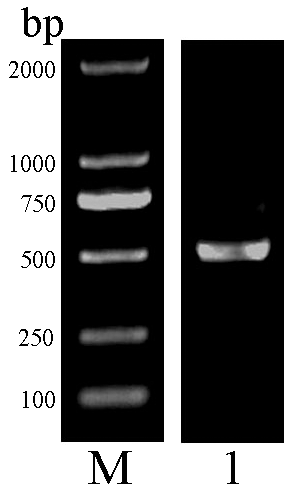

Supplement: Supplementary file 1 — Figure S1. PCR analyses confirming the association of O. sinensis with bacteria. M, DL2000 DNA marker. Lane 1, PCR amplicon of V4-V5 region of 16S rRNA. (TIF 38 kb) [file 12864_2019_5544_MOESM1_ESM.tif]

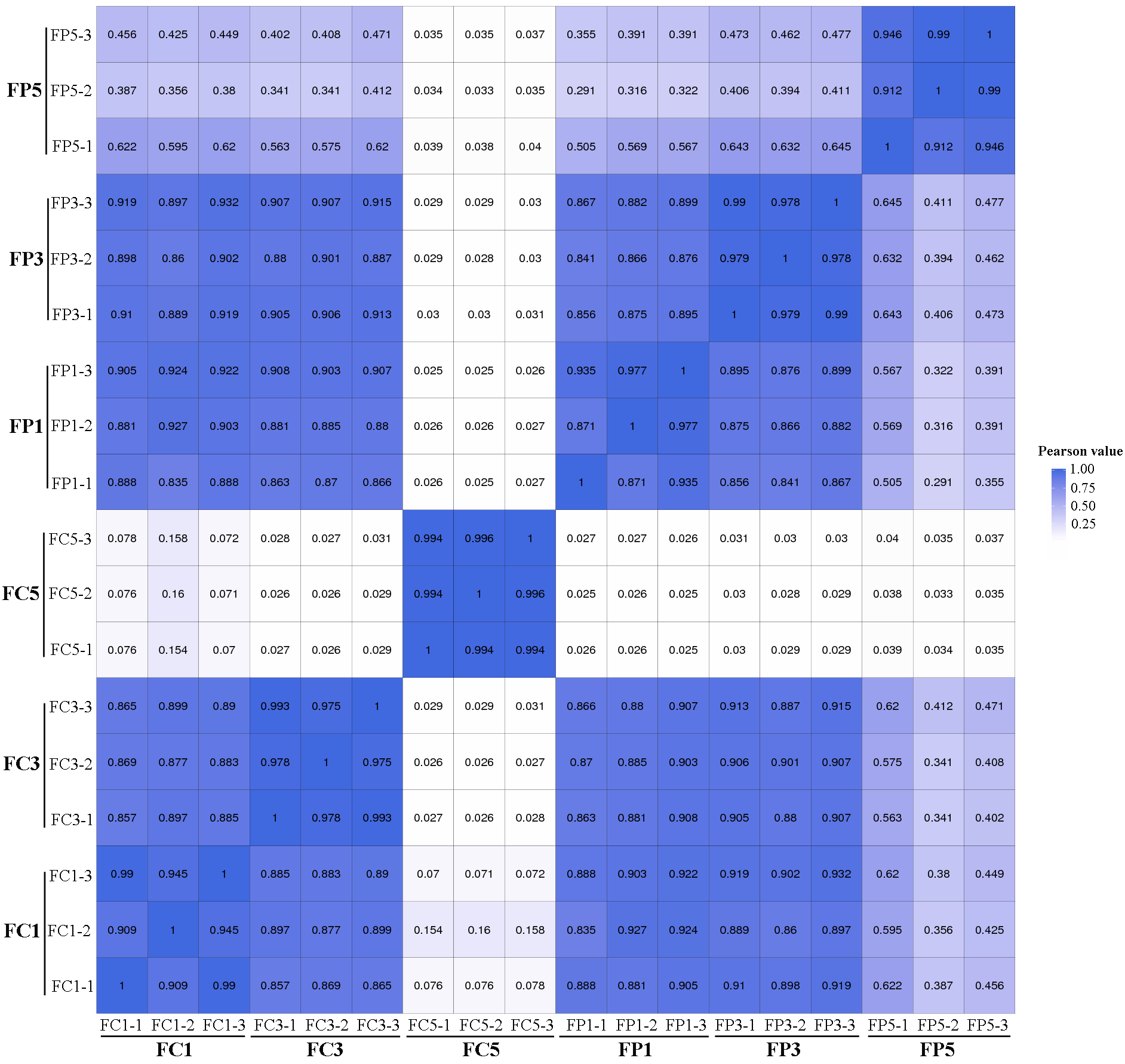

Supplement: Supplementary file 3 — Figure S2. Heatmap indicating the square of correlation value from three biological replicates. The correlation values were assessed by using the Pearson method. (TIF 476 kb) [file 12864_2019_5544_MOESM3_ESM.tif]
